# Use of D-Squame^®^ as a Minimally Invasive Technique to Evaluate Skin Immune Response Biomarkers in Canine Atopic Dermatitis †

**DOI:** 10.3390/vetsci12010004

**Published:** 2024-12-28

**Authors:** Marion Mosca, Nadège Milhau, Mélanie Legain, Adrien Idée, Xavier Langon, Didier Pin

**Affiliations:** 1 Interactions Cells Environment, 2016. A104, VetAgro Sup, Dermatology Department, 69280 Marcy L’étoile, France; nadege.milhau@vetagro-sup.fr (N.M.); melanie.legain@vetagro-sup.fr (M.L.); adrien.idee@anicura.fr (A.I.); didier.pin@vetagro-sup.fr (D.P.); 2 Research and Development Derm Research, Royal Canin, 30470 Aimargues, France; xavier.langon@royalcanin.com

**Keywords:** dog, cytokines, tape strips, ELISA, experimental model

## Abstract

Evaluation of skin inflammation biomarkers in canine atopic dermatitis (AD) currently requires skin biopsies. Tape stripping has been shown to be a reliable technique to study biomarkers in the stratum corneum (SC) in humans. The aim of this study was to assess the immune response and identify biomarkers in the SC of dogs with canine AD using D-squame^®^ as a minimally invasive technique. Eight beagle dogs were epicutaneously sensitized to *Dermatophagoides farinae* extract after tape stripping on sensitized site (S); twice a week for 49 days. Two sites were determined: lesional site (L) and non-lesional site (NL) on eight dogs affected spontaneously with AD. Adhesive tape strips D-Squame^®^ were applied on each site. Skin concentrations of 10 cytokines were analyzed with an ELISA kit. Our results revealed a significant increase of IL-13, IL-4, and TNF-α concentrations in S and L sites. Regarding IFN-γ, its concentration was significantly increased in L skin and increased but not significantly in S sites. All the alarmins were not differentially expressed except IL-33 in the S site. IL-31, IL-1β, and IL-10 were not detectable. D-squame^®^ seems to be a suitable technique to extract inflammatory cytokines from the SC of dogs, and IL-13, IL-4, TNF-α, and IFN-γ could be interesting biomarkers of canine AD.

## Simple Summary

Atopic dermatitis (AD) is a common dermatosis in humans and dogs. Traditional methods to study pathogenesis and biomarkers in canine AD required skin biopsies, but this study used minimally invasive adhesive tape (D-squame^®^) to collect samples from the skin’s outermost layer. Eight dogs were exposed to allergens over 49 days, and eight dogs were affected with AD spontaneously. Tape strips were applied to both affected and unaffected skin areas in each dog. The results showed higher levels of specific immune markers (IL-13, IL-4, TNF-α, and IFN-γ) in inflamed skin compared to unaffected areas. Some markers, like IL-31, IL-10, and IL-1β, were undetectable, and others, like IL-33, showed minor changes. These findings suggest that tape stripping is a reliable, painless way to measure inflammation in dogs with AD. Additionally, IL-13, IL-4, TNF-α, and IFN-γ could serve as key indicators for diagnosing and monitoring this condition.

## 1. Introduction

Atopic dermatitis is a common dermatosis affecting both humans and dogs, with a respective prevalence of 10% in adults to 20% in children, and 3 to 15% in canine [1,2,3]. The complex pathogenesis of this disease involves skin barrier defects, dysbiosis, and immune system responses. Many cytokinic pathways have been described and may be expressed differentially among patients. Some of these cytokines could serve as biomarkers.

Biomarkers have been defined as “characteristics that are objectively measured and evaluated as an indicator of normal biologic processes, pathogenic processes, or pharmacologic responses to a therapeutic intervention”, according to the National Institutes of Health (NIH) [4]. Seven different types of biomarkers have been defined by the FDA-NIH Biomarker Working Group: “susceptibility/risk, diagnostic, monitoring/severity, prognostic, predictive, pharmacodynamic/response, and safety” [5]. Thus, assessment of all these biomarkers could play a significant role in the diagnosis, prognosis, management, and treatment of AD.

Studies have explored relevant and validated biomarkers from blood samples and skin biopsies of canine AD or in dogs affected spontaneously with this dermatosis. Most studies have been conducted on blood samples and have evaluated diagnostic and severity biomarkers. Promising cytokines, differentially expressed in the blood of atopic dogs, are IL-13, IFN-γ, TNF-α, CCL28, IL-10, and TGF-β [6,7,8,9,10,11]. Phosphodiesterase 4D (PDE4D) gene expression in the blood was statistically higher in atopic dogs and could be used as a diagnostic biomarker [10,12]. Serum CCL17 concentrations represent a promising biomarker for disease severity and therapeutic response [13,14]. Some biomarkers for disease severity have been studied, such as IL-33 in the skin [15], S100-A8 in the skin and the serum [16,17], and IL-31 and CCL17 in the serum [8,14].

Tape stripping has been shown to be a reliable technique to study biomarkers in the stratum corneum (SC) in humans [18,19,20,21,22]. In dogs, one study determined the dimensions of corneocytes collected from healthy dogs and cats, and from dogs suffering from atopic dermatitis [23], and one study has shown that this tool could effectively remove the SC [24]. The aim of our study was to assess the immune response and identify biomarkers in the SC of dogs with canine AD (experimental model and spontaneously affected dogs) using this minimally invasive technique. The first step was to characterize cytokines from tape strips (TS) on an experimental model of canine AD. The second step was to use this method on dogs spontaneously affected with AD and presented at University referral consultations. Finally, a comparison of all the results was done to identify some reliable and pertinent biomarkers. Their identification could be useful as a future diagnostic tool or to have a more targeted treatment according to individual specific immune responses.

## 2. Materials and Methods

### 2.1. Ethical Considerations

The protocol of this study was submitted to the Ethics Committee of VetAgro Sup (French Ethical Committee number 18) and authorized by the French Ministry of Higher Education and Research under Project number APAFIS: 2245V2. All owners of dogs that were included prospectively signed a written consent form for participation in this study.

### 2.2. Animals

#### 2.2.1. Experimental Model of Canine AD

Eight healthy laboratory beagle dogs were included in this study. All dogs were males with a mean age of 38.4 months (34–40) and a mean weight of 10.3 kg (8.6–11.6). No history of previous dermatosis was reported. They were not genetically related to each other. They lived outside and were allocated in 2 open-air kennels of 40 m^2^ each that enabled dogs to move around freely and have interactions with each other. Sensitization was performed indoors twice a week for 7 weeks; each dog was kept in an individual kennel for 10 min after the application of the sensitized product on their back to prevent licking.

#### 2.2.2. Dogs Affected Spontaneously with AD

Dogs presented at the veterinary University hospital were included if they were diagnosed with canine AD for the first time. Diagnosis of canine AD was made on the basis of compatible history, clinical signs, and the presence of at least six positive criteria in the diagnostic criteria of Favrot [25]. Other pruritic dermatoses were excluded after combing and skin scraping. Secondary skin infections that often complicate AD were treated before the sampling date, which was verified by impression smears and cytological evaluation of acetate tape impression.

### 2.3. Study Design

#### 2.3.1. Epicutaneous Sensitization with *Dermatophagoides farinae*

**Figure 1:**
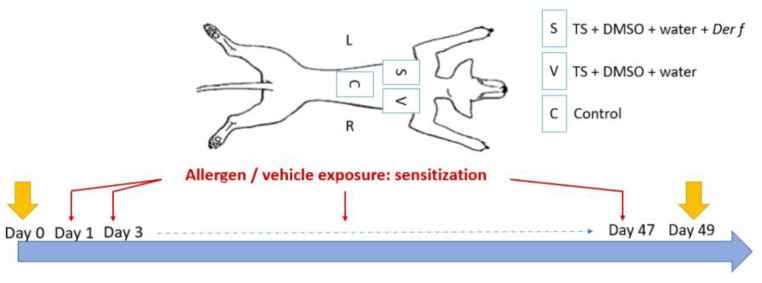
Three sites were defined on the back of Beagle dogs. The control site (C) did not receive any treatment, the vehicle site (V) received DMSO and water after tape stripping (TS) with commercial adhesive tape, and the sensitized site (S) received *Dermatophagoides farinae* (Der f), DMSO, and water after tape stripping. Applications were made twice a week for 49 days. D-squames were performed at Day 0 and at Day 49.

The sensitization protocol was based on a previous study [26]. Three areas of 3 × 3 cm were defined and clipped on the back of each dog. The first area, corresponding to the control site (C), did not receive any treatment and was kept safe as a control for healthy skin throughout the study duration. The two other areas were stripped by successive tape strips with commercial adhesive tape (Transparent Tape Scotch^®^, 19 mm; 3M France, Cergy-Pontoise, France) twice a week for 49 days. Tape stripping was stopped when the skin became erythematous and shiny in order to remove most of the SC. Then, the sensitized site (S) received a 500 µL solution composed of *Dermatophagoides farinae* (Der f) house dust mite (Citeq Biologics, Groningue, The Netherlands), 200 μg of protein per square centimeter diluted in dimethysulfoxide (DMSO, Sigma-Aldrich, Saint-Louis, MI, USA) 75% and water 25% (Sterile Otec water) twice a week directly after the strips. The vehicle site (V) received only DMSO and water but no allergen protein (Figure 1). The total quantity of the solution was progressively applied on the sites so that the liquid was absorbed uniformly by the skin. These quantities were chosen according to a previously described protocol for a murine model of AD [27]. The skin was squeezed with fingers placed on each line delimiting the sites (length of a rectangle) and placed in a digital caliper (Electronic digital caliper DIN 862, RS Pro, Manutan, Gonesse, France). The caliper was tightened with an equivalent pressure for each measurement [26].

#### 2.3.2. Stratum Corneum Sample Collection

Circular adhesive tapes (22 mm diameter, 3.8 cm^2^, D-squame^®^ discs; Monaderm, Monaco, France) were placed and pressed for 5 s with a pressure of 225 g·cm-^2^, using a D-Squame Pressure Instrument D500 (Monaderm, Monaco). For the experimental model, D-squames were placed on the control site (C) at T0 and on the vehicle (V) site and the sensitized (S) site at T1. Several tapes were applied until obtaining a shiny aspect of the skin as previously described [24].

**Figure 2:**
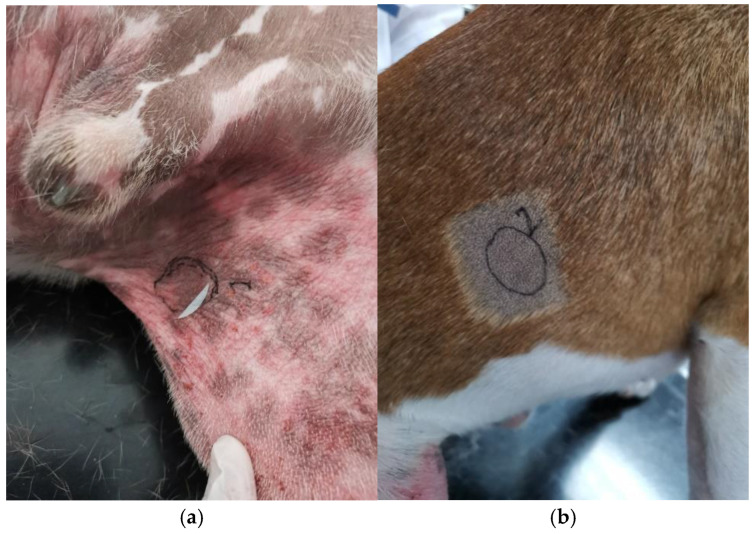
(**a**) Lesional site (S) noted 1 on the photo with erythema, alopecia, and lichenification on the inguinal area and (**b**) non-lesional site (NL) noted 2 on the photo on the right flank of a Staffordshire Bull terrier.

In dogs affected spontaneously with canine AD, samples were collected from the lesional (L) and non-lesional (NL) sites, after clipping gently these areas (Figure 2). L sites were characterized by eczematous lesions and were sometimes already shiny. We thus collected 25 strips from every dog to standardize the samples.

Protein content on each tape was determined using Squame Scan 850A (Monaderm, Monaco, France) optical density (OD) measures for total protein. Each tape was scanned by the instrument, and optical absorption at 850 nm was measured. Each disc was immersed in phosphate-buffered saline containing 0.05% Tween 20 (PBS Tween) (Thermo scientific, Waltham, MA, USA) and stored in a cryotube at 4 °C for 24 h maximum.

#### 2.3.3. Cytokines Analysis

For each sampling site, the first three tapes were removed. For the other tapes, soluble proteins were extracted from each tape by ultrasonication (15 min) in an ultrasound sonification bath (Bransonic M2800-E Sigma-Aldrich, Missouri, USA) with 0.8 mL PBS Tween in ice water, and then pooled. The final dilution volume was the same for each dog and site. Total soluble protein content was determined using Pierce Micro BCA protein assay kit (Thermo Fisher Scientific, Rockford, IL, USA) according to the manufacturer’s instructions. Concentrations of cytokines were corrected by the total surface of samples (one disk is a 22 mm diameter circle corresponding to a 38.01 mm^2^ surface) in pg·mL^−1^·cm^−2^. Skin concentrations of 10 cytokines: IFN-γ (DuoSet^®^ ELISA Canine IFN-γ Immunoassay, R&D systems, Minnesota, USA), IL-1β (Canine IL-1 beta ELISA Kit, Invitrogen, Massachusetts, USA), IL-4 (DuoSet^®^ ELISA Canine IL-4, R&D systems, Minnesota, USA), IL-10 (DuoSet^®^ ELISA Canine IL-10 Immunoassay, R&D systems, Minnesota, USA), IL-13 (Canine IL13 ELISA Kit, orb437164, Biorbyt, Durham, USA), IL-33 (Dog IL33 ELISA Kit, orb403474, Biorbyt, Durham, USA), TNF-α (DuoSet^®^ ELISA Canine TNF-α Immunoassay, CATA00, R&D systems, Minnesota, USA), TSLP (Canine TSLP ELISA kit, CT03630, Kendal Scientific, Lincolnshire, UK), IL-25 (Canine IL-25 ELISA kit, MBS049988, MyBioSource, San diego, USA), and IL-31 (canine IL-31 ELISA kit, CI0041, Kendal scientific, Lincolnshire, UK and dog IL-31 ELISA kit, abx259000, Abbexa, Cambridge, UK) were determined using the manufacturer’s instructions for each kit. Vials were pooled and the final dilution volume was the same for each dog and site. Analyses were performed in duplicate on the same plaque except for IL-31. For statistical analysis, cytokine concentrations below the detection limit, but within the fit curve range, were taken unchanged, and cytokine concentrations below the fit curve range were assigned half the value of the lowest sample concentration below the detection limit to maintain the ranking order [21,28,29].

### 2.4. Statistical Methods

Statistical analyses were performed using Prism 9 (GraphPad Software, La Jolla, CA, USA). Data distribution was tested with the Shapiro–Wilk normality test. In the canine experimental model of AD, multiple comparisons were made using a Friedman test. Correction for multiple testing, using the Benjamini–Hochberg method was conducted for all the resulting *p*-values. For dogs with spontaneous AD, the comparison between L and NL sites was performed using a paired *t*-test for normal distributed values and Wilcoxon’s signed rank test otherwise. *p*-values < 0.05 were considered significant.

## 3. Results

### 3.1. Animals

Eight dogs with spontaneous AD were included. There were two females and six males, three Staffordshire bull terriers, two French bulldogs, one American Staffordshire terrier, one American bully pocket, and one Bernese Mountain dog. The mean age was 16.1 months (6–34) and the mean weight was 20.1 kg (9–46.3).

### 3.2. Sensitization of the Experimental Model of Canine AD

**Figure 3:**
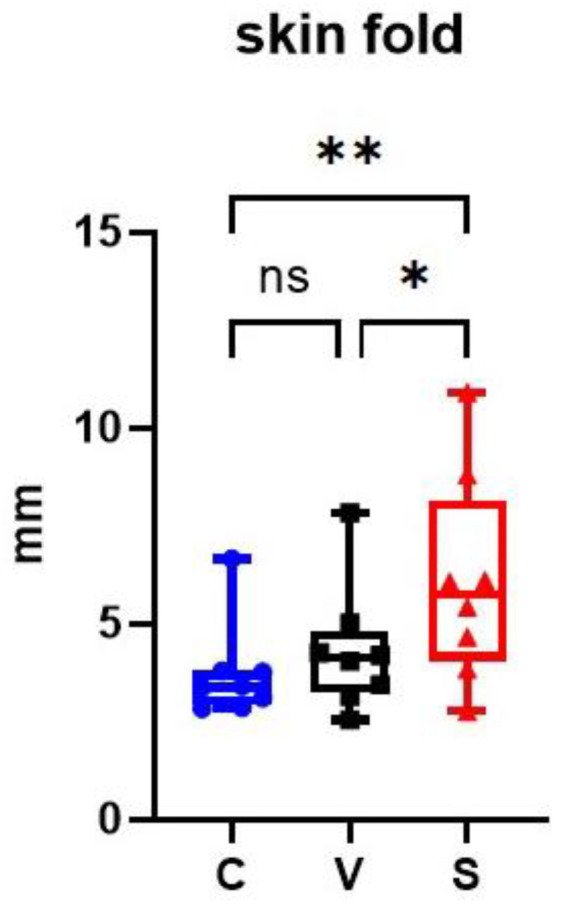
Representation of skin fold thickness in mm on the three sites of the experimental model of canine AD. The sensitized site (S) showed a significant increase in skin fold thickness measurements compared with the control site (C) and the vehicle site (V). * *p* < 0.05, ** *p* < 0.001, ns: non significant.

At the end of the sensitization, V and S sites had an increased average skin fold thickness compared with the C site. Skin fold in the S site was significantly increased compared with the C and V sites (*p* = 0.0028 and *p* = 0.0128 respectively); there was no significant difference between the C and V sites (*p* = 0.1586) (Figure 3).

### 3.3. Total Amount of Proteins and Total Amount of Soluble Proteins

#### 3.3.1. Total Amount of Proteins

**Figure 4:**
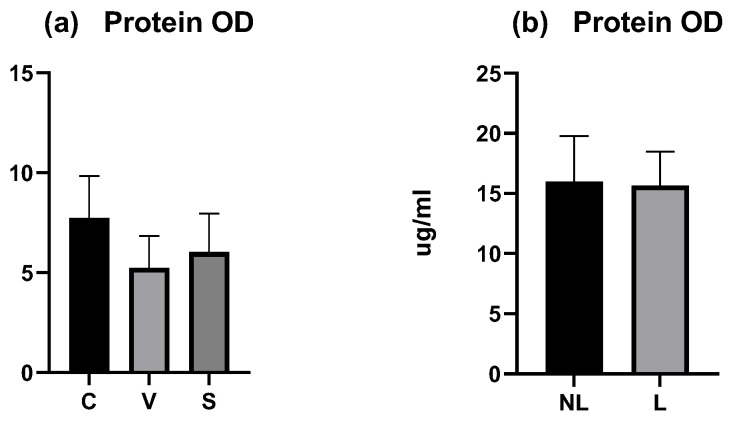
OD values measured by squame scan. (**a**) Total mean amount of proteins on C, V, and S sites were not significantly different. (**b**) Total mean amount of proteins on NL and L sites were not significantly different. C = control site, V = vehicle site, S = sensitized site, NL = nonlesional site, L = lesional site.

The total amount of proteins was measured on each tape for each dog and the mean (calculated from all tapes except the first three) was compared between sites. In the experimental model of canine AD, the mean OD was similar between the three sites and did not differ significantly (*p* > 0.05) (Figure 4a). The coefficient of variation was low with 27.26% for C data, 30.37% for V data, and 32.05% for S data.

In dogs with spontaneous AD, the mean total protein OD was similar between the NL and L sites and did not differ significantly (*p* > 0.05) (Figure 4b). The coefficient of variation was low with 23.9% for NL data and 18.07% for L data.

These results showed that protein OD was not representative of inflammation and the variation between individuals was low.

**Figure 5:**
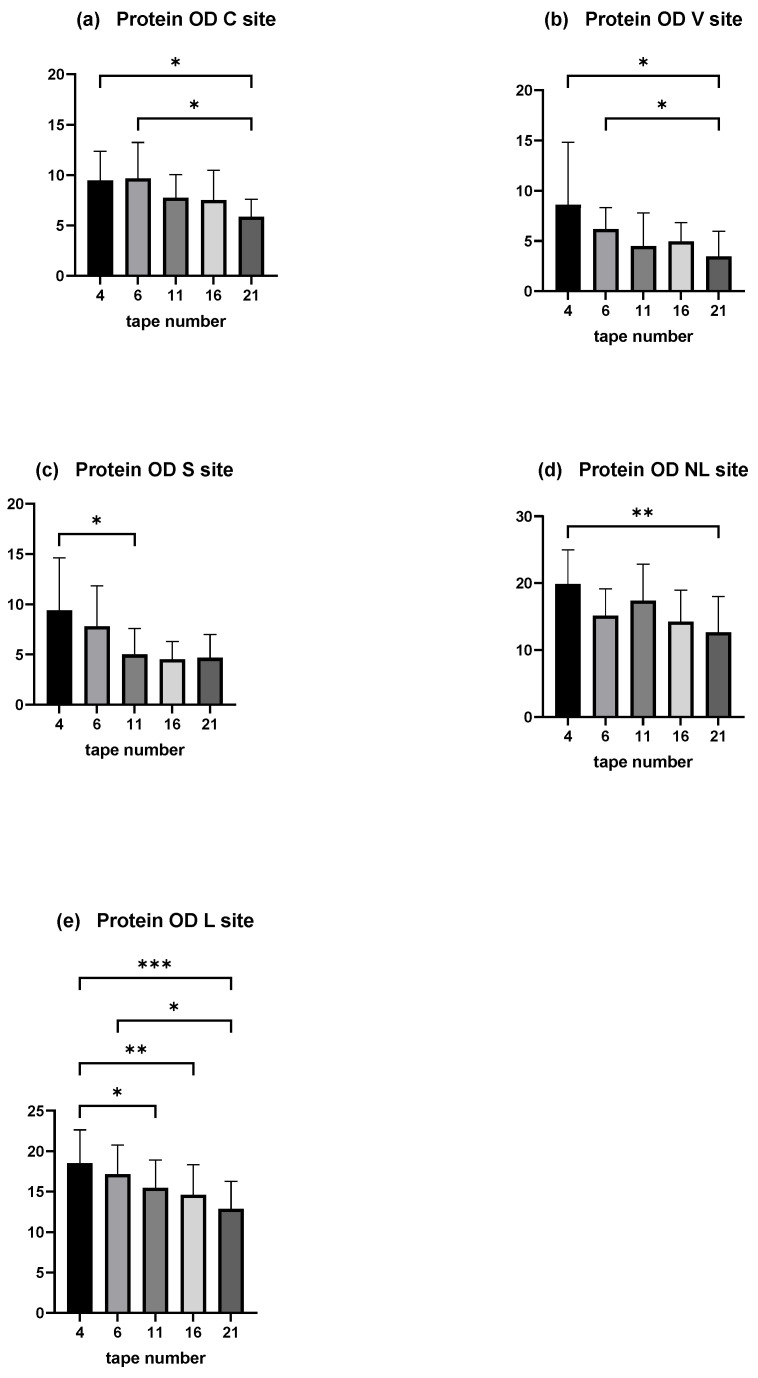
OD values measured by squame scan, showing decreasing amount of total protein through the depths of stratum corneum. (**a**) is the C site, (**b**) the V site, and (**c**) the S site of the experimental model of canine AD. (**d**) is the NL site and (**e**) the L site of dogs affected spontaneously with canine AD. The deeper tape contains significantly less amount of proteins when compared with the two first near the surface except in the S site. The five columns represent tape no. 4, tape no. 6, tape no. 11, tape no. 16, and tape no. 21. * *p* < 0.05, ** *p* < 0.001, *** *p* < 0.0001.

Moreover, the amount of total protein on each tape decreased with depth of sampling. A significant decrease was shown between the mean protein OD of tape numbers 4 and 6 compared with the deepest (tape number 21) in the C and V sites (*p* < 0.05). The decrease was significant comparing the mean protein OD of tape numbers 4 and 11 in the S site (Figure 5a–c).

In dogs affected spontaneously with AD, a decrease with depth is observed, significantly between disc numbers 4 and 21 in the non-lesional site, and between discs numbers 4 and 11, 4 and 16, 4 and 21, and 6 and 21 (Figure 5d,e).

The variation of total protein OD was similar between the experimental model and dogs affected spontaneously with AD. Since the number of discs was different among dogs, these results motivated the pooling of the samples.

#### 3.3.2. Total Amount of Soluble Proteins

**Figure 6:**
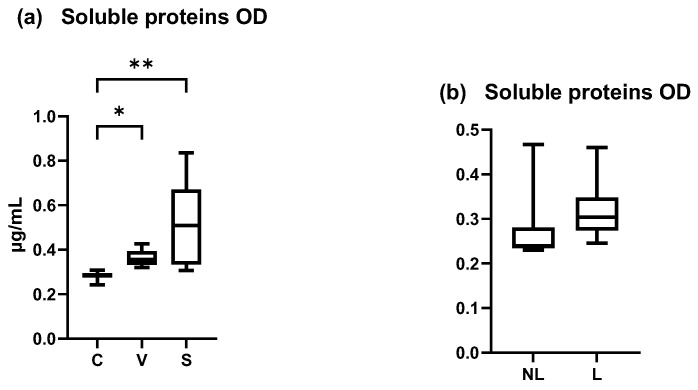
OD values measured by Pierce BCA protein. (**a**) Total mean amount of soluble proteins were significantly increased in S site compared with C and V site (*p* < 0.05). (**b**) Total mean amount of soluble proteins on NL and L sites were not significantly different. C = control site, V = vehicle site, S = sensitized site, NL = nonlesional site, L = lesional site. * *p* < 0.05, ** *p* < 0.001.

The total amount of soluble proteins in our samples increased between the C and V, C and S, and V and S sites. A significant increase was shown between the C and S sites and C and V sites but not between the V and S sites (Figure 6a) (Friedman test *p* = 0.0024, *p* = 0.0478 and *p* = 0.1479, respectively). The coefficient of variation was 32.03% for C data, 27.10% for V data, and 63.85% for S data.

In dogs spontaneously affected with AD, the total amount of soluble proteins was non-significantly different between NL and L (Wilcoxon matched-pairs signed rank test, *p* > 0.05) (Figure 6b). The coefficient of variation was 151.7% in NL data and 64.05% in L data.

These results showed an increase of the amount of soluble proteins in inflamed sites, that was not homogeneous between individuals and explain why these data were not chosen as a normalizer for cytokine concentrations. Concentration was normalized with the total area of D-squames^®^ extraction of SC (surface = 3.8 cm^2^).

### 3.4. Cytokine Analysis

#### 3.4.1. Canine Experimental Model of AD (Figure 7)

##### Innate Immune Responses (Alarmins)

**Figure 7:**
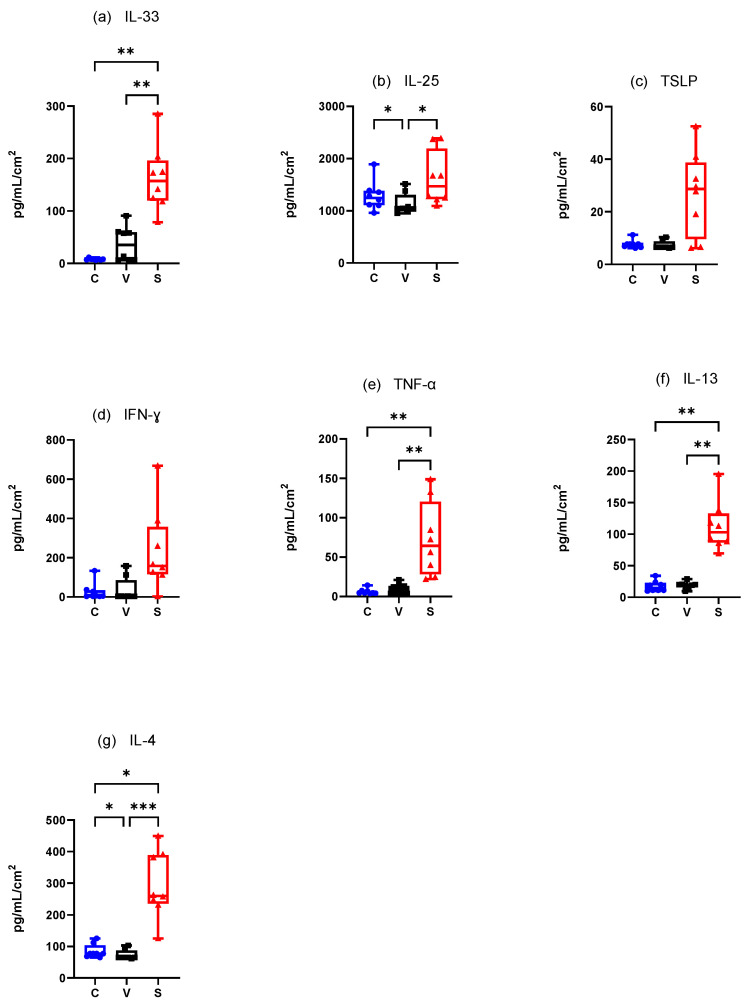
Mean concentration expression of (**a**) IL-33, (**b**) IL-25, (**c**) TSLP, (**d**) IFN-γ, (**e**) TNF-α, (**f**) IL-13, and (**g**) IL-4 on C, V, and S sites in canine model of AD. C = control site, V = vehicle site, S = sensitized site. * *p* < 0.05, ** *p* < 0.001, *** *p* < 0.0001.

Mean IL-33 concentration·cm-^2^ was significantly higher in the S site compared with the C sites (*p* = 0.0012) and V sites (*p* = 0.0031) but did not significantly differ between the C and V sites (*p* = 0.2160) (Figure 7a).

Mean IL-25 concentration.cm-^2^ was significantly increased in the S sites compared to the V sites (*p* = 0.0261) and in the C sites compared with V sites (*p* = 0.0478) (Figure 7b). Mean TSLP concentration was increased in the S sites but TSLP did not differ significantly in any site (C vs. V *p* = 0.2736, C vs. S *p* = 0.2736, and V vs. S *p* = 0.0770) (Figure 7c).

##### Adaptative Immune Responses (Th1)

There was an increase of mean IFN-γ concentration·cm-^2^ in the S site but the differences between the sites were not significant (C vs. V *p* = 0.3332, C vs. S *p* = 0.3332, V vs. S *p* = 0.1433) (Figure 7d). Mean TNF-α concentration.cm-^2^ was significantly higher in the S site compared with the C site (*p* = 0.0031 and V site (*p* = 0.0012). There was no significant difference between the C and V sites (*p* = 0.2160) (Figure 7e).

##### Adaptative Immune Responses (Th2)

There was a significant increase in mean IL-13 concentration·cm-^2^ between the C and S sites and the V and S sites (*p* = 0.004 for both) but not between the C and V sites (*p* > 0.99) (Figure 7f). Mean IL-4 concentration.cm-^2^ was significantly higher in the S site compared with the C site (*p* = 0.0128) and the V site (*p* = 0.0002) and was significantly higher in the C site compared with the V site (*p* = 0.0468) (Figure 7g).

#### 3.4.2. Dogs Spontaneously Affected with Canine AD (Figure 8)

##### Innate Immune Responses (Alarmins)

**Figure 8:**
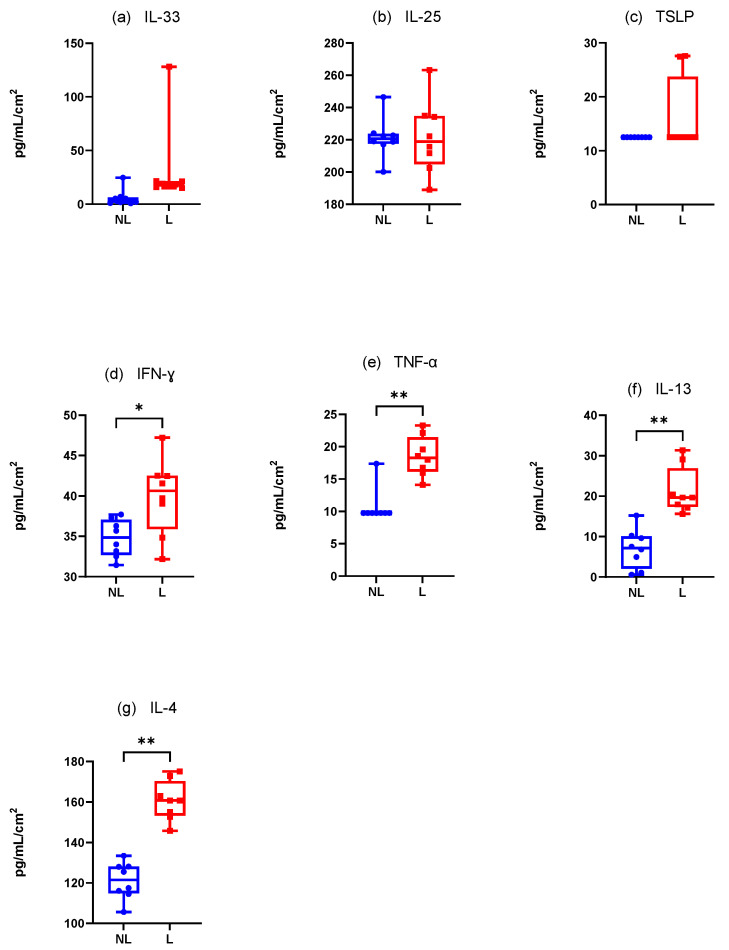
Mean concentration expression of (**a**) IL-33, (**b**) IL-25, (**c**) TSLP, (**d**) IFN-γ, (**e**) TNF-α, (**f**) IL-13, and (**g**) IL-4 on L and NL sites in dogs spontaneously affected with AD. NL = nonlesional site, L= lesional site. * *p* < 0.05, ** *p* < 0.001.

There was a non-significantly increase in the mean IL-33 concentration·cm-^2^ between the NL and L sites. Furthermore, only one value appeared detectable with the ELISA kit (Figure 8a). The results were the same for TSLP with only three dogs with detectable values on the L site (Figure 8b). Finally, IL-25 was not differently assessed in the NL and L sites (Wilcoxon test, *p* = 0.7344) but every value was close to the maximum value detectable on the ELISA kit (Figure 8c).

##### Adaptative Immune Responses (Th1)

There was a significant increase in the mean IFN-γ and TNF-α concentration·cm-^2^ in the L sites compared with the NL sites (Wilcoxon test, *p* = 0.0078 and *p* = 0.0039, respectively) (Figure 8d,e).

##### Adaptative Immune Responses (Th2)

There was a significant increase in the mean IL-4 and IL-13 concentrations·cm-^2^ in the L sites compared with the NL sites (Wilcoxon test, *p* = 0.004 for both) (Figure 8f,g).

IL-31, IL-1β, and IL-10 were not detectable with our experimental conditions.

Considering all the results in the experimental model and in dogs spontaneously affected with atopic dermatitis, some similarities have been shown for TH2 cytokines with a significant increase of IL-4 and IL-13 in sensitized and lesional sites compared with control sites. TH1 cytokines were significantly increased in the S and L sites compared with control sites except for IFN-γ, for which the increase was not statistically significant in the experimental model. Among alarmins, only IL-33 was statistically increased in S and L sites compared with control sites in the experimental model.

#### 3.4.3. Ratio of Increase

**Figure 9:**
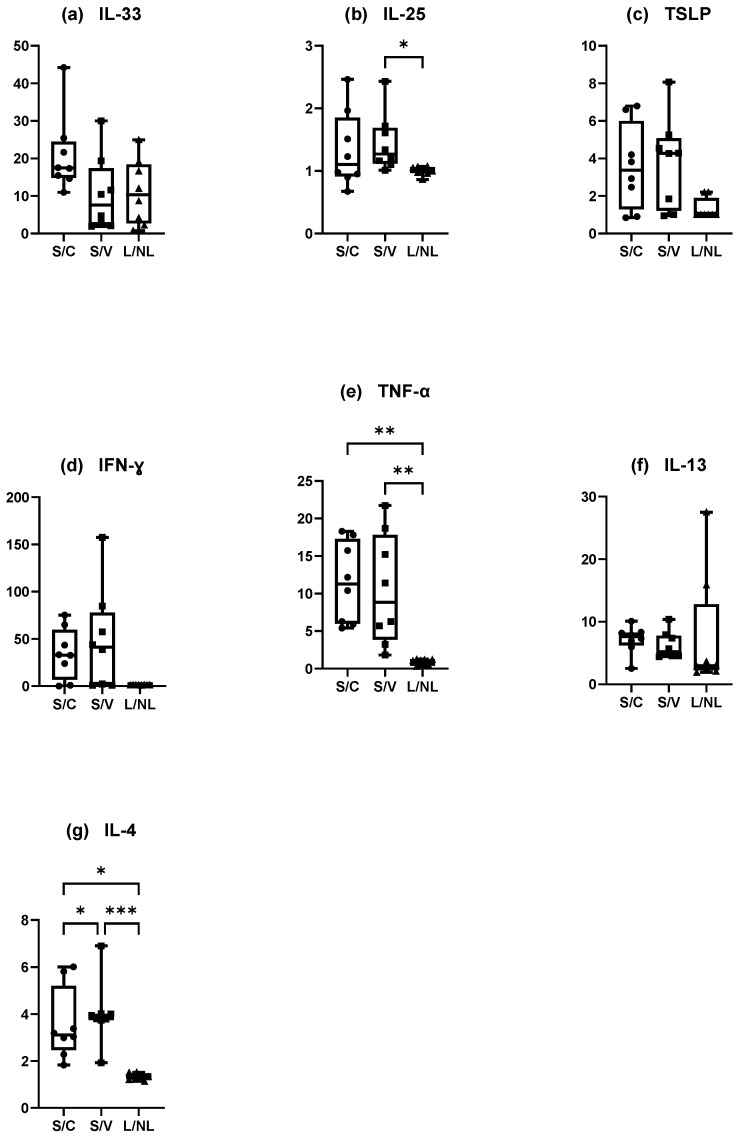
Ratio of increase of cytokines (**a**) IL-33, (**b**) IL-25, (**c**) TSLP, (**d**) IFN-γ, (**e**) TNF-α, (**f**) IL-13 and (**g**) IL-4 in S and L sites. Graphs represent the normalized concentration of one cytokine on S/L site divided by the normalized concentration of this cytokine in C or V/NL site for the experimental model of canine AD/dogs affected spontaneously with canine AD. C = control site, V = vehicle site, S = sensitized site, NL = nonlesional site, L = lesional site. * *p* < 0.05, ** *p* < 0.001, *** *p* < 0.0001.

Results of the ratio between concentrations of cytokines on the L sites divided by those on the NL or S sites divided by those on the C or V sites are presented (Figure 9). The higher the result, the more difference was present between the sites. The results showed the close concentration of IL-4, TNF-α, IFN-γ, and IL-25 in the L and NL sites in dogs spontaneously affected with AD (ratio close to 1). In the experimental model, there was a higher difference between cytokines present in the S sites compared with the C or V sites except for IL-25.

### 3.5. Effects on Dogs

All dogs tolerated D-squames^®^ application well; a few got impatient due to the contention time associated with sampling on lesional sites (axillary or inguinal regions). A slight erythema was sometimes associated with the region of tape stripping but disappeared spontaneously in approximatively 24 h.

## 4. Discussion

This study showed that D-squames^®^ appeared to be a suitable technique for studying proteins from dog skin.

The choice of a standardized pressure, number of strips, time of application, and sonication were made based on previous studies. In fact, the barrier disruption level has been shown to be influenced by pressure, time, and anatomical location [30], which justifies our choice to use D-Squame Pressure Instrument D500. The number of successive tapes was determined in accordance with the results of a previous study [24], but dogs affected with AD already had a shiny aspect of the skin, considered the deeper limit of the SC, and it was chosen to standardize the number of strips to 25 instead [24]. This could represent a bias because the depth of sampling may vary from dog to dog. There is no significant difference in protein yield between pressure times of 5 or 10 s, determined by both BCA and Squame Scan in a study [31]; thus pressure time was set to 5 s to expedite sampling. Many studies calibrated a sonication time of 15 min [21,32,33] even if a sonication time of 10 and 15 min were shown to have the same yield [31].

The total amount of protein, as evaluated by Squame Scan 850A, was not different between sites and did not increase after sensitization or in lesional sites. However, when focusing on soluble proteins, the total amount is increased at these sites. This could be explained by the fact that other proteins (keratins, adhesion proteins, for example) that are not associated with inflammation are extracted by D-squame^®^. Therefore, this data does not seem to be relevant for characterizing AD in dogs.

The total amount of proteins has previously been shown to decrease with depth in the SC whereas the amount of soluble proteins is stable [21,31,34]. We showed similar results regarding the amount of total proteins that were decreasing with depth of sampling except in the S sites of the experimental model where the decrease was not significant between tape number 4 and the deepest one, number 21. This could be explained by the low number of dogs in our study and the fact that the protein OD value is quite variable between two successive tapes even if a decrease is measurable. Further studies are necessary to determine the amount of soluble proteins according to the depth of the SC to compare with total protein amounts. It has been shown that OD measurements decreased significantly (between 50–90%) after sonication [31] but that no differential expression was shown for several cytokines and antimicrobial peptides with depth in soluble proteins [21,35]. In human AD, previous reports concluded that only one tape could be enough to represent the inflammation molecules in the SC. As we did not study this aspect, we chose to pool our samples and normalize with the surface and not with the total soluble proteins. Furthermore, the concentration of soluble proteins was significantly increased in the S sites and lower in the C sites, so normalization based on this data would have reversed the reality.

The variation coefficient showed a close amount of total proteins regardless of the individual, corroborating our results, and a close amount of total soluble proteins except for the S sites. This seems in concordance with the results of other studies that rely on this factor and not the total amount of proteins and could be explained by the high individual variability of response to epicutaneous sensitization to *Dermatophagoides farinae* [26,36].

We investigated levels of TH2 cytokines, IL-4 and IL-13, and TH1 cytokines, IFN-γ and TNF-α, as well as alarmins, IL-33, TSLP, and IL-25. Previous studies evaluated the importance of these cytokines in spontaneous and experimental canine AD by measuring concentration in circulating blood, assessing the mRNA in skin biopsies and blood, and by immunohistochemistry or immunostaining on skin biopsies [8].

Our results revealed a significant increase of IL-13 and IL-4 concentrations in the S sites compared with the C sites in the experimental model of canine AD and in the L sites compared with NL in dogs spontaneously affected with AD. These results correlate with those previously published in experimental models of AD with ELISA tests [36], and with a significant increase in the expression of IL-13 mRNA after the challenge in the skin [37,38]. In the blood of canine AD experimental models, IL-4 mRNA level was increased [39] or stable [37,38]. In dogs spontaneously affected with AD, results in peripheral blood showed an increase in IL-13 concentrations [11,40] and a similar level of IL-4 compared with healthy dogs [11,41]. The results in the skin were similar with increased Il-13 and IL-4 expressions [7,40,42,43]. One study could not detect mRNA expression of IL-4 in dog atopic skin [41].

Our study revealed a significant increase in the concentration of TNF-α in both the S and L sites. Regarding IFN-γ, its concentration was significantly increased in L skin compared with NL skin and increased but not significantly in the S sites compared with the C and V sites. Similarly, higher levels of mRNA of IFN-γ and TNF-α in atopic lesional skin have been detected in atopic dogs with semi-quantitative RT-PCR [40,41,44]. Another study showed that mRNA of IFN-γ was significantly amplified most frequently from chronic, lichenified lesional skin and TNF-α was increased but not significantly in samples of lesional skin compared with samples of NL skin [42]. In peripheral blood, TNF-α concentrations were increased in AD dogs [11]. Two studies showed different results in the experimental model of canine AD with increased mRNA expression of IFN-γ and decreased mRNA expression of TNF-α in the skin [37] and a significant increase of IFN-γ in the blood [36].

Alarmin results showed a significant increase of IL-33 concentrations in the S sites compared with the C sites. Other alarmins were increased but not significantly in the S sites compared with the C and V sites, and in the L sites compared with the NL sites except for IL-25, for which levels were similar. Similarly, in an experimental model of AD, one study found a significant upregulation of genes encoding IL-33, 48 h after house dust mite challenges [7]. Nevertheless, in the skin of dogs spontaneously affected with AD, mRNA levels of IL-33 in lesional sites were higher than those in the normal skin of healthy dogs and were associated with chronic lesions in contrast with our results [45]. A statistically significant difference in IL-33 immunostaining level was shown in lesional skin compared to non-lesional skin and increased with the chronicity of the lesions [15]. TSLP levels were assessed from skin biopsies in one study and results showed significantly increased TSLP expressions in the L and NL sites of dogs with canine AD compared with healthy control dogs, but there was no difference between the L and NL sites [46], as in our results. Finally, no study had evaluated IL-25 expression in spontaneous and experimental canine AD but one study used RT-PCR to reveal upregulation of IL-25 gene expression in periostin-stimulated keratinocytes [47].

The evolution of cytokine concentrations in our model is close to what has already been described, as we found similar results for cytokine levels in lesional skin compared to non-lesional skin of dogs spontaneously affected with AD except for IL-33. D-squames^®^ appear suitable to extract cytokines from the SC of dogs, and these results show similarities with those obtained from skin biopsies and blood samples.

The results of the ratio of cytokine concentrations showed for the first time the possibility of inflammation in the SC of non-lesional skin of dogs spontaneously affected with AD using a minimally invasive technique. In the experimental model, we did not observe this effect due to the control site being completely normal and the vehicle site not being sensitized to *Dermatophagoides farinae* but instead receiving physical stress (tape stripping) and irritation with DMSO. Nevertheless, IL-4, TNF-α, IFN-γ, and IL-25 levels were similar in the L and S sites, although IFN-γ and TNF-α had OD that were under the detection limit for all dogs in the NL sites. These results suggest the existence of skin inflammation in the non-lesional skin of dogs spontaneously affected with AD.

The experimental model of canine AD was performed based on a previous study [26]. This study checked validity with skin inflammation monitoring (clinical evaluation, skin fold thickness, histological lesion severity score, and epidermis thickness measurements on histologic samples), IgE and IDR tests, flow cytometry to look after T cell populations, and proliferation *t* test [26]. Our results found the reproducibility of the model regarding skin fold thickness measurement that seems a good marker for sensitization. The model seemed even more reliable with our results since the results of the S sites were similar to the results of the L sites compared with the NL or C sites.

A major limitation of our study is the low number of dogs, which prevents the generalization of our results. Further studies with more dogs are needed to confirm our findings. Levels of soluble proteins were very low, and many cytokines had an OD under the detection limit. For example, levels of IL-13 and IL-4 were low, with values below the detection limit in most samples from the C and V sites, as well as for IL-13 expression in the NL skin. In the C, V, and NL sites, TNF-α concentrations were also below the detection limit. Furthermore, we were not able to detect IL-31, IL-10, and IL-1β with the ELISA kits we used in our experimental conditions. This could be due to the low levels of soluble proteins we were able to extract or the high detection limit of the kits we used. Considering the crucial role of IL-31 in canine AD, its increase in the blood of atopic dogs [48,49,50], and its correlation with the severity of lesions in dogs spontaneously affected with AD [51] and in experimental models [49], further studies are necessary to better evaluate its presence in the SC of dogs. Finally, it would have been interesting to add skin biopsies in our study to compare our results with histopathological examination. This could have allowed for quantification of cytokines using D-squame, which would be relevant for diagnosis, as well as for assessing cell location and profile to better understand the pathophysiology of AD.

## 5. Conclusions

D-squame appears to be a good technique for extracting inflammatory cytokines from the SC of dogs. However, it also proved to be time-consuming technique and labor-intensive in the lab. In both the canine experimental model of AD and in dogs spontaneously affected with AD, this technique shows that IL-13, IL-4, TNF-α, and IFN-γ could serve as interesting biomarkers, with significantly increased expression in the SC of sensitized sites and lesional skin. Limitations of this study include the low quantities of proteins extracted from the tapes and the technical difficulty of performing ELISA with these samples. Further studies are necessary to confirm these results, improve the technique, and evaluate the correlation between these biomarkers, clinical lesion scores, and therapeutic management.

## Data Availability

Raw data are available upon reasonable request to the corresponding author.
